# Effects of nitrogen fertilizers on the bacterial community diversity and the weathering of purple mudstone in Southwest China

**DOI:** 10.3389/fmicb.2023.1164826

**Published:** 2023-06-29

**Authors:** Chunpei Li, Maopan Fan, Xuan Wang, Xue Li, Guang Zhao, Gangcai Liu, Jixia Zhao

**Affiliations:** ^1^College of Resources and Environment, Yunnan Agricultural University, Kunming, China; ^2^Key Laboratory of Mountain Surface Processes and Ecological Regulation, Chinese Academy of Sciences, Institute of Mountain Hazards and Environment, Chinese Academy of Sciences, and Ministry of Water Conservancy, Chengdu, China; ^3^University of the Chinese Academy of Sciences, Chinese Academy of Sciences, Beijing, China; ^4^Key Laboratory of Ecosystem Network Observation and Modeling, Institute of Geographic Sciences and Natural Resources Research, Chinese Academy of Sciences, Beijing, China

**Keywords:** purple mudstone weathering, bacterial community structure, urea, ammonium bicarbonate, weathering indices

## Abstract

**Introduction:**

Rock weathering is crucial in the development of soil. Yet the role of bacteria in the fine particle-forming process of purple mudstone is not fully understood, especially under nitrogen fertilization.

**Methods:**

In this study, the particles (0.25 mm to 1 mm) of purple mudstone from Penglai Group (J_3_p) were selected as the test material. Two nitrogen fertilizers, i.e., urea (U) and ammonium bicarbonate (AB), and four application levels (0, 280, 560, and 840 N kg∙ha^−1^) with 18 replications were designed in an incubation experiment. The weathering indices and bacterial community structure of the purple mudstone particles were investigated after 120 days of incubation.

**Results:**

The results showed that the weathering indices of purple mudstone particles in the AB treatment were higher than that in the U treatment at the same fertilization levels and a reducing trend was observed with increasing nitrogen fertilizer levels under the same nitrogen fertilizer application types. The diversities of the bacterial community were extremely significantly altered by nitrogen fertilizer application (*p* < 0.01). The effect of the nitrogen fertilizer application level on the beta diversity of the bacterial community (*R^2^* = 0.34) was greater than that of the nitrogen fertilizer application type (*R^2^* = 0.20). Through stepwise regression analysis, the positive effects of nitrification of Nitrobacter (*Nitrolancea*) (*R^2^* = 0.36), the Phosphorous-dissolving bacteria (*Massilia*) (*R^2^* = 0.12), and N-NO_3_^−^ (*R^2^* = 0.35) on the weathering indices of J_3_p purple mudstone particles could be observed. Structural equation modelling indicated that nitrogen fertilizer application level affects the abundance of the dominant species at the genus level (*Nitrolancea* and *Massilia*), and key environmental factor (N-NO_3_^−^), which in turn accelerated the weathering indices (59%).

**Discussion and Conclusion:**

Our findings imply that the enhancements of nitrification of Nitrobacter (*Nitrolancea*) and of phosphorus solubilization of Phosphorous-dissolving bacteria (*Massilia*) by nitrogen fertilization are the key factors affecting the weathering indices of J_3_p purple mudstone particles.

## 1. Introduction

Weathering of parent rock is an integral part of the Earth’s surface processes of soil formation and plays a critical role in maintaining terrestrial ecosystems (Dixon et al., 2012; Zhu et al., 2013; Israeli et al., 2021), as well as controlling the long-term chemical composition of groundwaters, rivers, lakes, and oceans (Duan et al., 2002; Godderis et al., 2009). The weathering of rocks has long been a research focus in geosciences, as it also plays a critical role in the global carbon cycle (Li and Elderfield, 2013; Torres et al., 2016) and in regulating atmospheric CO_2_ on geological time scales (Them et al., 2017). It has even been suggested that enhanced weathering would curb present-day anthropogenic carbon emissions (Xu and Liu, 2010; Torres et al., 2016; Strefler et al., 2018; Beerling et al., 2020). As the key process of soil formation, weathering of parent rocks is an aspect that cannot be ignored in influencing global climate change.

The weathering rate of rock is affected by both natural (e.g., geological settings and topographical conditions, the provenance of source rock, temperature, precipitation, and chemical composition) and artificial factors (Köhler et al., 2003; Kirschbaum et al., 2005; Caspari et al., 2006; Chetelat et al., 2008; Huang et al., 2013; Moore et al., 2013). Mass balance calculations indicated that the contribution of anthropogenic factors to parent rock weathering accounts for 16–40% (Liu et al., 2018). Analyzing the hydrochemistry in regional rivers indicated that nitric acid and sulfuric acid produced by anthropogenic activities promote the weathering of minerals (Yu et al., 2016) and increase the weathering rate of carbonate rocks by approximately 6% (Zhu et al., 2019). Some studies indicated that exogenous H^+^ addition induces clay minerals dissolving, i.e., Montmorillonite, Kaolinite, Mica, and Illite (Tombácz and Szekeres, 2006; Zhang et al., 2009; Sokolova, 2013; Bibi et al., 2014), while more difficult-to-dissolve minerals are retained, i.e., Quartz.

Fertilization, an artificial disturbance factor in agricultural production activities, is becoming increasingly prominent, and will affect soil and other resources, including the impact on the weathering and soil formation of the parent rock. Nitrogen fertilizer is the most commonly used fertilizer, accounting for almost 50% of fertilizer consumption, and the amount of nitrogen application in China accounts for 32% of the world at present (Yu et al., 2019). According to the National Statistical Yearbook, the amount of nitrogen fertilizer applied in China increased from 93.4 kg∙ha^−1^ in 1980 to 153.0 kg∙ha^−1^ in 2018. The long-term large-scale or excessive use of nitrogen fertilizer application, especially ammonium nitrogen fertilizer, can cause soil acidification (Chao et al., 2011; Goulding, 2016; Yang et al., 2016), which is widely known to affect the weathering of parent rock (Zhao et al., 2021). Furthermore, the application of nitrogen fertilizers has a critical effect on soil microbial processes, key microbial community structures, and N availability (Zeng et al., 2015; Gai et al., 2016). Previous studies also implied nitrification of fertilizer nitrogen could prominently promote the weathering of minerals (Pacheco et al., 2013). Although microbial action is one of the most significant factors affecting the weathering of rocks and minerals (Krumbein, 1983), the alterations of bacterial community structure and pH on the weathering of parent rocks driven by nitrogen fertilizer addition is not fully understood yet.

Purple rock is a sedimentary rock, mainly distributed in the upper Yangtze River in China, especially in Sichuan and Yunnan provinces. The coverage of purple soil in these two provinces accounts for over 75% of the national purple soil area, of which Sichuan Province accounts for 51.5% and Yunnan Province accounts for 23.4% (Liu, 2008). Due to its fast physical weathering, purple rock is easily broken into rock particles or gravel by environmental factors as well as anthropogenic activities, and crops can be planted directly on weathered products. The 1 mm particle formation process was dominated by physical and chemical weathering. However, biological weathering was enhanced in the <1 mm particles formation process. Soil nutrients derived from purple rocks are largely from the weathering processes, especially P- and K-rich minerals. Purple rock is generally considered the nutrient source for purple soil (Gao et al., 1999; He, 2003). The purple rock area has practical significance in food and ecological security in China. N fertilization is a major practice in crop production in this area. Long-term application of nitrogen fertilizer causes acidification of purple soil (Huang, 2012). A number of previous studies have been carried out on physical and chemical weathering (Gao et al., 1999; Zhang et al., 2017; Zhao et al., 2021), but relatively few studies have been reported on the effects of biological weathering on purple rocks under N application.

In order to understand the bacterial effects driven by nitrogen fertilizer application on the fine particle formation of purple mudstone particles, an indoor incubation experiment was carried out in this study. Our aim was to provide information for purple soil formation under current agricultural production.

## 2. Materials and methods

### 2.1. Rock sample collection

The purple mudstone of the Penglai Group (J_3_p) (31°16′N, 105°27′E), one of the most widely distributed alkaline purple parent rocks in the Sichuan Basin (He, 2003), was collected from a rock layer 60 cm below the earth surface without anthropogenic influences. This was then air-dried to a constant weight at room temperature and mechanically broken. The particles with a particle size of 0.25-1 mm were selected with the main components of clay minerals and main oxide, presented in Supplementary Table S1 and Supplementary Table S2, respectively.

### 2.2. Experimental design

Urea (U, 46%) and ammonium bicarbonate (AB, 17%), as the main N fertilizers applied in the study area, were selected. Four nitrogen fertilizer addition levels (0, 280, 560, and 840 N kg∙ha^−1^ described as CK, 100% CL, 200% CL, and 300% CL) were used, of which the 280 N kg∙ha^−1^ is the conventional application level. Specifically, seven treatments were used in the incubation experiment: (1) CK: no fertilizers, (2) U1: urea of 280 N kg∙ha^−1^, (3) U2: urea of 560 N kg∙ha^−1^, (4) U3: urea of 840 N kg∙ha^−1^, (5) AB1: ammonium bicarbonate of 280 N kg∙ha^−1^, (6) AB2: ammonium bicarbonate of 560 N kg∙ha^−1^, and (7) AB3: ammonium bicarbonate of 840 N kg∙ha^−1^. Each treatment was replicated 18 times.

The incubation experiment was conducted as follows. After weighing, 100 g J_3_p of mudstone particles (0.25-1 mm) were placed into 240 mL culture bottles. These culture bottles were put in an incubator with a constant temperature of 25°C and soil moisture was maintained at 40% of the field water capacity for 7 days as the preparation phase. Then, relevant rate and already dissolved nitrogen fertilizer was added into the culture bottles according to the treatments, while soil moisture content was adjusted to 60% ~ 70% of field water capacity. This incubation condition was maintained during the whole experiment and the whole cultivation process lasted for 120 days.

### 2.3. Sampling of J_3_p mudstone particles

Sampling was conducted on the 1st, 7th, 15th, 30th, 60th, and 120th days from the start of the incubation. Each time, three culture bottles were randomly selected from 18 replicates of each treatment. After sampling, fresh samples were used to determine the dissolved organic carbon (DOC), total dissolved nitrogen (TDN), N-NH_4_^+^, N-NO_2_^−^, and N-NO_3_^−^. Samples collected on day 120 were also used to analyze the bacterial community (samples kept at −4°C) and the physical and chemical properties (samples air-dried under normal temperature).

### 2.4. Measurement of indicators

The dissolved organic carbon (DOC), total dissolved nitrogen (TDN), N-NH_4_^+^, and N-NO_3_^−^ were measured using a continuous flow analytical system (AutoAnalyzer 3) (Wang et al., 2007; Yin et al., 2015). The content of dissolved organic carbon and nitrogen in purple mudstone particles after 120 days of culture with nitrogen fertilizer was measured by methods of agrochemical analysis of soil (Lu, 1999) and is shown in Table 1. The pH was measured in deionized water at a ratio of 1:2.5 (soil: water) using an air-dried sample by a pH meter (Sartorius PB-10). After the lithium metaborate melt, the content of SiO_2_, Al_2_O_3_, Fe_2_O_3_, MgO, Na_2_O, K_2_O, and CaO of minerals was determined by inductively coupled plasma atomic emission spectrometry (ICP-AES), and this test was carried out in Chengdu Baihui Biotechnology Co. The mineral composition and the clay mineral content were measured by an X-ray diffractometer (XD-3010301) (Zhang and Fan, 2003). The mechanical composition was determined by the screening method (Liu, 1996), and the field capacity of the purple rock weathering product was measured by the bubble water method (Xu, 2007). Moreover, the water-soluble Ca^2+^ was measured in deionized water at a ratio of 1:10 (soil: water) by atomic absorption spectrophotometers.

**Table 1:** The content of dissolved organic carbon and nitrogen in purple mudstone particles after 120 days of culture.

Nitrogen treatment	pH_H2O_	DOC g∙kg^−1^	TDN g∙kg^−1^	N-NH_4_^+^ g∙kg^−1^	N-NO_3_^−^ g∙kg^−1^
CK	9.11 ± 0.02b	0.45 ± 0.03d	0.03 ± 0.00 g	0.01 ± 0.00d	0.02 ± 0.00d
U1	8.03 ± 0.02c	0.60 ± 0.00c	1.13 ± 0.01e	0.04 ± 0.00d	1.01 ± 0.00a
U2	9.38 ± 0.01a	0.27 ± 0.01e	2.26 ± 0.06b	1.55 ± 0.08b	0.05 ± 0.01d
U3	9.41 ± 0.01a	1.95 ± 0.03a	2.64 ± 0.03a	2.37 ± 0.04a	0.10 ± 0.01c
AB1	7.88 ± 0.05d	1.20 ± 0.02b	1.01 ± 0.07f	0.05 ± 0.00d	0.92 ± 0.03b
AB2	9.40 ± 0.02a	0.34 ± 0.01de	1.25 ± 0.02d	1.20 ± 0.02c	0.05 ± 0.01d
AB3	9.43 ± 0.01a	0.60 ± 0.10c	1.57 ± 0.01c	1.54 ± 0.01b	0.03 ± 0.00d

The no fertilizers (CK), urea of 280 N kg∙ha−1 (U1), urea of 560 N kg∙ha−1 (U2), urea of 840 N kg∙ha−1 (U3), ammonium bicarbonate of 280 N kg∙ha−1 (AB1), ammonium bicarbonate of 560 N kg∙ha−1 (AB2), and ammonium bicarbonate of 840 N kg∙ha−1 (AB3) were observed. And the pH, dissolved organic carbon (DOC), total dissolved nitrogen (TDN), N-NH4+, and N-NO3− concentrations after 120 days of nitrogen fertilizer application were displayed in Table 1. Numbers depict means ± standard deviations (*n* = 3), the same below. ANOVA was conducted followed by the Duncan test for multiple comparisons, and different lowercase letters indicate that there were significant differences among all treatments (*p* < 0.05).

### 2.5. DNA extraction, PCR amplification, and sequencing

According to the manufacturer’s protocol, bacterial DNA samples were extracted from 0.5 g fresh samples using the Fast DNA SPIN extraction kit (MP Biomedicals, Santa Ana, CA, United States). The V3-V4 region of the bacterial 16S rRNA gene was amplified (upstream primers 338F 5’-ACTCCTACGGGAGGCAGCA-3′ and the downstream 806R 5′- GGACTACHVGGGTWTCTAAT −3′) by PCR. Specifically, sample-specific 7-bp barcodes were incorporated into the primers for multiplex sequencing. The PCR components contained 5 μL of Q5 reaction buffer (5×), 5 μL of Q5 High-Fidelity GC buffer (5×), 0.25 μL of Q5 High-Fidelity DNA Polymerase (5 U/μl), 2 μL (2.5 mM) of dNTPs, 1 μL (10 uM) of each Forward and Reverse primer, 2 μL of DNA Template, and 8.75 μL of ddH_2_O. Thermal cycling consisted of initial denaturation at 98°C for 2 min, followed by 25 cycles consisting of denaturation at 98°C for 15 s, annealing at 55°C for 30 s, and extension at 72°C for 30 s, with a final extension of 5 min at 72°C. PCR amplicons were purified with Agencourt AMPure Beads (Beckman Coulter, Indianapolis, IN) and quantified using the PicoGreen dsDNA Assay Kit (Invitrogen, Carlsbad, CA, United States). After the quantification step, applicants of PCR were pooled in equal amounts, and the sequencing was performed using the Novaseq-PE250 pattern of the Illumina MiSeq platform at Shanghai Personal Biotechnology Co., Ltd. (Shanghai, China).

### 2.6. Evaluation index of the rocks weathering

The chemical index of alteration (*CIA*-Nesbitt and Young, 1982), chemical index of weathering (*CIW*- Harnois, 1988), chemical proxy of alteration (*CPA*- Buggle et al., 2011), and modified *CIA* (*CIX*-Garzanti et al., 2014) were widely applied to evaluate the weathering of the rocks. Ion composition ratios of the minerals were used to calculate *CIA*, *CIW*, *CPA*, and *CIX* by the following formula:


$$CIA=Al_{2}O_{3}/\left(Al_{2}O_{3}+Na_{2}O+K_{2}O+CaO^{*}\right)\times 100\cdots \cdots \mathrm{Formula}\;1$$

$$CIW=Al_{2}O_{3}/\left(Al_{2}O_{3}+Na_{2}O+CaO^{*}\right)\times 100\cdots \cdots \mathrm{Formula}\;2$$

$$CPA=Al_{2}O_{3}/\left(Al_{2}O_{3}+Na_{2}O+\right)\times 100\cdots \cdots \mathrm{Formula}\;3$$

$$CIX=Al_{2}O_{3}/\left(Al_{2}O_{3}+Na_{2}O+K_{2}O\right)\times 100\cdots \cdots \mathrm{Formula}\;4$$

where $\;CaO^{*}$ in formula 1, 2, and 3 is the amount of CaO contained in the silicate fraction of the rocks.

### 2.7. Data statistics analysis

Microbiome bioinformatics was performed with QIIME 22019.4 (Bolyen et al., 2019) with slight modifications according to the official tutorials.^1^ Raw sequence data were demultiplexed using the demux plugin followed by primers cutting with the cutadapt plugin (Martin, 2011). Sequences were then quality filtered, denoised, merged, and chimera removed using the DADA2 plugin (Callahan et al., 2016). Non-singleton amplicon sequence variants (ASVs) were aligned with MAFFT (Katoh et al., 2002) and used to construct a phylogeny with fasttree2 (Price et al., 2009). Alpha-diversities metrics (Chao1, Shannon, and Simpson) and beta-diversity metrics were estimated using the diversity plugin. Taxonomy was assigned to ASVs using the classify-sklearn naïve Bayes taxonomy classifier in a feature-classifier plugin (Bokulich et al., 2018) against the Greengenes 13_8 99% OTUs reference sequences (McDonald et al., 2012). Based on the result of the ASV taxonomic unit processed by the method of DATA2 (Callahan et al., 2016), the relative abundance of the bacterial composition at the genus level was counted to explore the dominant bacterial species under the different nitrogen fertilizer treatments, and the graph of the bacterial composition at the genus level was drawn by Origin 2021. The differences in diversities (alpha and beta diversities) of the bacterial community were analyzed under different nitrogen fertilizer treatments based on Bray Curtis, permutational multivariate analysis of the variance (PERMANOVA) (Mcardle and Anderson, 2001), and nonmetric multidimensional scaling (NMDS) of Bray Curtis. One-way ANOVA was applied to the data by the Duncan method *via* IBM SPSS Statistics 25 to compare the differences in bacterial alpha-diversities and physicochemical indices among the nitrogen fertilizer treatments.

In addition, the multiple linear stepwise regression analysis was applied to measure the effectiveness of the bacterial dominant species and the physicochemical indices on weathering indices. Based on the result of the multiple linear stepwise regression analysis, the partial least squares structural equation modelling (PLS-SEM) was used to construct structural equation models by SmartPLS 3 tools. The variance inflation factor (VIF), coefficient of determination (*R*^2^), and normed fit index (NFI) were applied to evaluate the model (Liu et al., 2022).

### 2.8. Accession numbers

The raw sequencing data from 16S rRNA genes were deposited in NCBI’s sequence read archive under the accession number PRJNA881222.^2^

## 3. Results

### 3.1. The content of dissolved organic carbon and nitrogen in purple mudstone particles after 120 days of culture

After 120 days of incubation, the pH (except for the 100% CL treatment), DOC (except for the 200% CL treatment), TDN, N-NH_4_^+^, and N-NO_3_^−^ were significantly more enhanced by nitrogen fertilizer addition than the CK treatment (*p* < 0.05). As seen in Table 1, for the same nitrogen fertilizer type, the pH, TDN, and N-NH_4_^+^ increased whilst the N-NO_3_^−^ decreased with increasing nitrogen fertilizer application. The DOC at U treatments displayed an increasing trend but a decreasing trend was observed at AB treatments with increasing nitrogen fertilizer addition. Additionally, under the same nitrogen fertilizer application levels, compared with the U treatment, the pH and N-NO_3_^−^ of the AB treatment were increased by −1.87% ~ 0.28% and − 73.33% ~ 7.14%, respectively, and the increase rate shows a trend of increasing first and then decreasing with the increasing of nitrogen fertilizer application. The N-NH_4_^+^ and DOC under the AB treatment were enhanced by 69.28% ~ 99.45% and − 35.25% ~ 33.33% compared with the U treatment, respectively, and the decrease rate displayed a decreasing trend with increasing of nitrogen fertilizer application. The TDN of the AB treatment was reduced by 10.36% ~ 44.69% compared with the U treatment, and the decrease rate displayed decreasing first and then an increasing trend with increasing nitrogen fertilizer application.

### 3.2. Nitrogen fertilizer addition changes bacterial community composition

**Figure 1:**
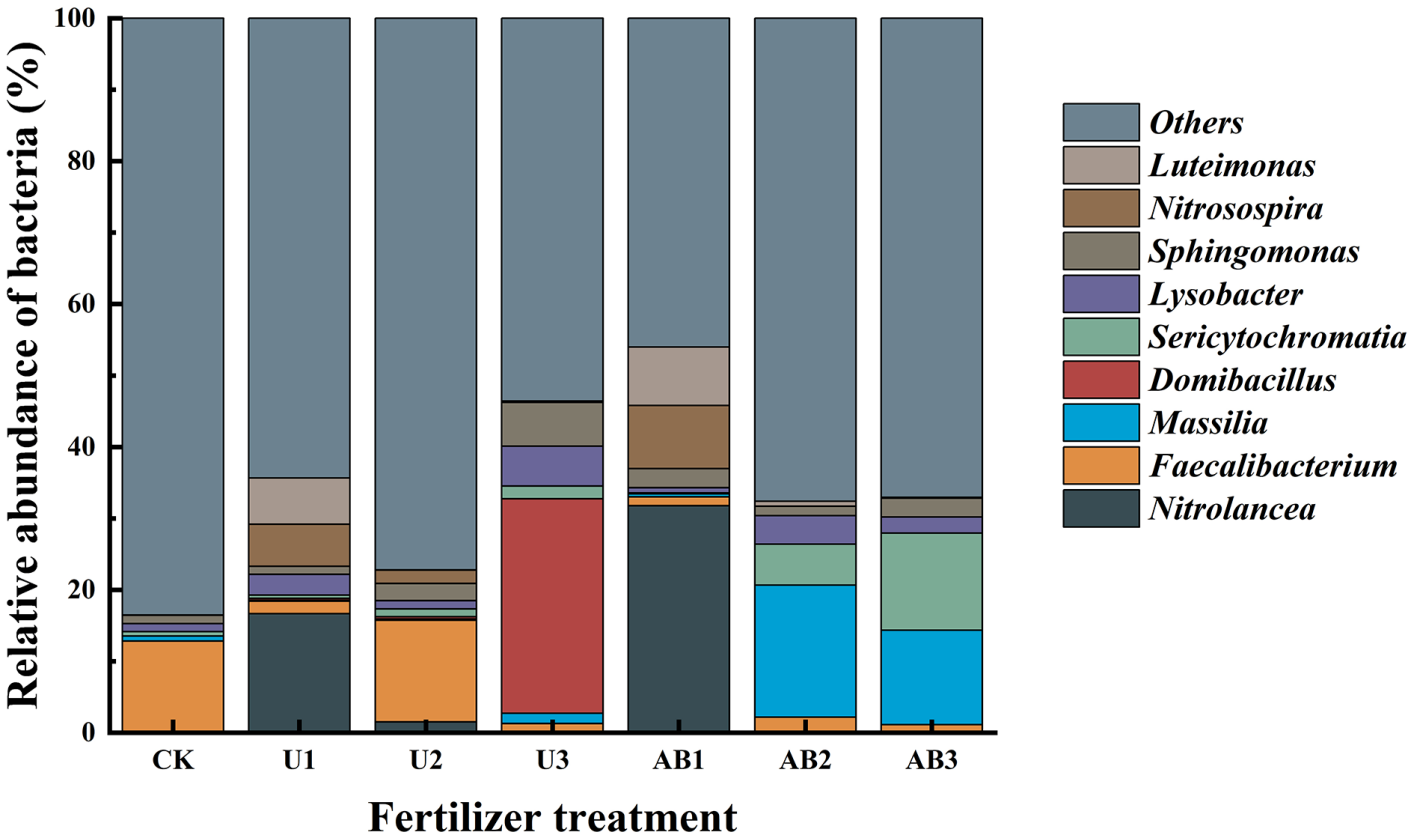
Relative abundances of the bacterial genera in J_3_p purple mudstone particles after 120 days of nitrogen fertilizer addition in the laboratory. Displayed is a bacterial genus that made up the top 10 of relative abundance; moreover, other relative abundance of bacterial were grouped as “Others.” The no fertilizers (CK), urea of 280 N kg∙ha^−1^ (U1), urea of 560 N kg∙ha^−1^ (U2), urea of 840 N kg∙ha^−1^ (U3), ammonium bicarbonate of 280 N kg∙ha^−1^ (AB1), ammonium bicarbonate of 560 N kg∙ha^−1^ (AB2), and ammonium bicarbonate of 840 N kg∙ha^−1^ (AB3) were observed.

After 120 days of incubation, the bacterial community composition (Figure 1) and the dominant species (Supplementary Figure S1) were measured under different nitrogen fertilizer applications at the genus level. Among all samples, the nine most abundant phylum of bacteria were *Nitrolancea* (7.14%), *Faecalibacterium* (4.94%), *Massilia* (4.94%), *Domibacillus* (4.38%), *Sericytochromatia* (3.35%), *Lysobacter* (2.53%), *Sphingomonas* (2.50%), *Nitrosospira* (2.39%), and *Luteimonas* (2.23%). Compared with CK treatment, the relative abundance of the *Nitrolancea*, *Nitrosospira*, and *Luteimonas* at U1 treatment were significantly enhanced (*p* < 0.05), but other main bacterial species had no significant difference (*p* > 0.05); the relative abundance of the *Domibacillus*, *Lysobacter*, and *Sphingomonas* at U3 treatment were significantly increased but the relative abundance of the *Faecalibacterium* was significantly decreased by 90.15% (*p* < 0.05), and other main bacterial species had no significant difference (*p* > 0.05); the relative abundance of the *Nitrolancea*, *Nitrosospira*, and *Luteimonas* at AB1 treatment were significantly increased but the relative abundance of the *Faecalibacterium* was significantly decreased by 90.32% (*p* < 0.05), and other main bacterial species had no significant difference (*p* > 0.05); the relative abundance of the *Massilia*, *Sericytochromatia*, and *Lysobacter* at AB2 treatment were significantly enhanced but the relative abundance of the *Faecalibacterium* was significantly decreased by 83.16% (*p* < 0.05), and other main bacterial species had no significant difference (*p* > 0.05); the relative abundance of the *Massilia* and *Sericytochromatia* at AB3 treatment were significantly enhanced but the relative abundance of the *Faecalibacterium* was significantly decreased by 91.15% (*p* < 0.05), and other main bacterial species had no significant difference (*p* > 0.05). Moreover, the relative abundance of bacteria was not significantly altered between U2 and CK treatment.

Under U fertilizer application treatment, the relative abundance of *Domibacillus* was observed to have an increasing trend but the relative abundance of *Nitrolancea*, *Nitrosospira*, and *Luteimonas* displayed a decreasing trend with the nitrogen fertilizer application levels increasing. Additionally, the relative abundance of *Faecalibacterium*, *Massilia*, *Sericytochromatia*, *Lysobacter*, and *Sphingomonas* displayed a non-linear characteristic. Under AB fertilizer application treatment, the relative abundance of *Sericytochromatia* displayed an increasing trend but the relative abundance of *Nitrolancea*, *Domibacillus*, *Nitrosospira*, and *Luteimonas* displayed a decreasing trend with the nitrogen fertilizer application levels increasing. Additionally, the relative abundance of *Faecalibacterium*, *Massilia*, *Lysobacter*, and *Sphingomonas* displayed a non-linear characteristic.

### 3.3. The difference in the bacterial community diversity under nitrogen fertilizer application

**Figure 2:**
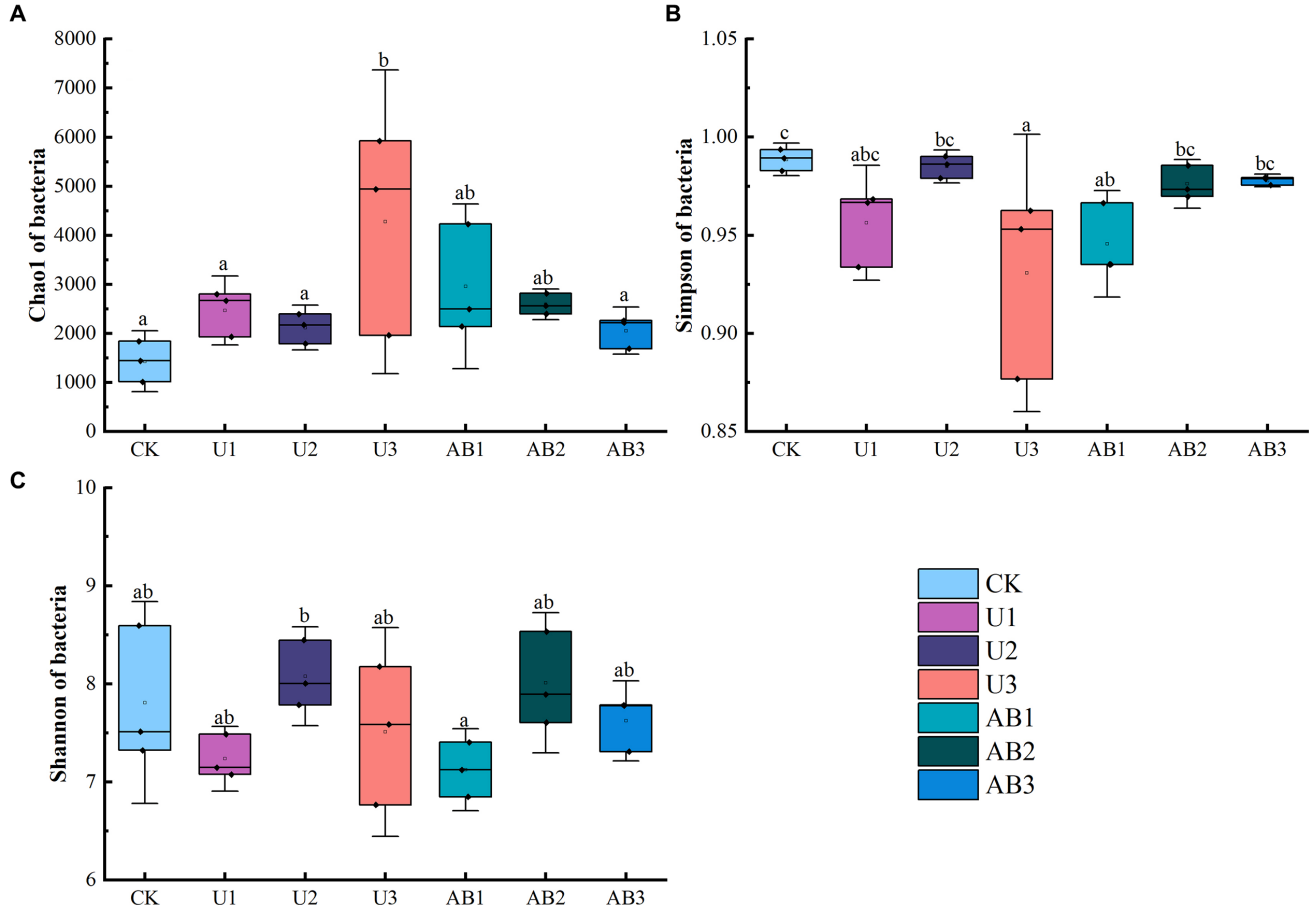
The Chao1 **(A)**, Simpson **(B)**, and Shannon **(C)** of the bacterial community after 120 days of nitrogen fertilizer addition in the laboratory. ANOVA was conducted followed by the Duncan test for multiple comparisons. Different lowercase letters indicate that there were significant differences among all treatments (*p* < 0.05). The no fertilizers (CK), urea of 280 N kg∙ha^−1^ (U1), urea of 560 N kg∙ha^−1^ (U2), urea of 840 N kg∙ha^−1^ (U3), ammonium bicarbonate of 280 N kg∙ha^−1^ (AB1), ammonium bicarbonate of 560 N kg∙ha^−1^ (AB2), and ammonium bicarbonate of 840 N kg∙ha^−1^ (AB3) were observed.

**Figure 3:**
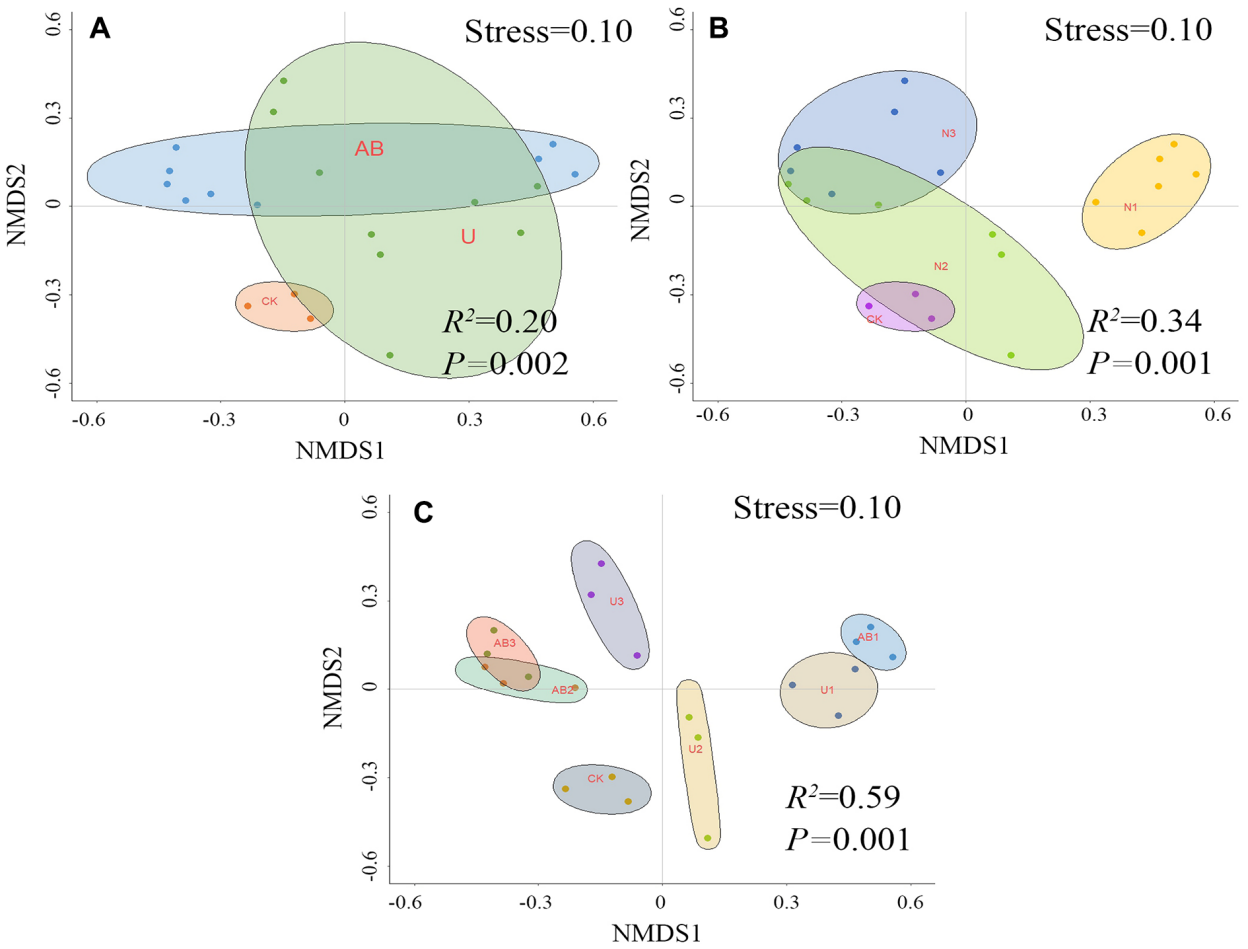
The beta diversity was calculated based on nonmetric multidimensional scaling (NMDS) after 120 days of nitrogen fertilizer addition in the laboratory **(C)**, and the bacterial beta diversity was restricted by the nitrogen fertilizer types **(A)** and levels **(B)** *via* permutational multivariate analysis of variance. The no fertilizers (CK), urea of 280 N kg∙ha^−1^ (U1), urea of 560 N kg∙ha^−1^ (U2), urea of 840 N kg∙ha^−1^ (U3), ammonium bicarbonate of 280 N kg∙ha^−1^ (AB1), ammonium bicarbonate of 560 N kg∙ha^−1^ (AB2), and ammonium bicarbonate of 840 N kg∙ha^−1^ (AB3) were observed.

After 120 days of incubation, nitrogen fertilizer application significantly altered the alpha and beta diversities of the bacterial community, and the effect of the nitrogen fertilizer addition levels on the alpha and beta diversities of the bacterial community was greater than the effect of the nitrogen fertilizer addition types (Figures 2, 3). The Chao1 of bacteria community under U1 and U3 treatment were enhanced by 72.15 and 101.65% compared to CK treatments, respectively, but other treatments (including U2, AB1, AB2, and AB3) decreased by 12.30 to 30.84% compared to CK treatments. The Shannon of the bacterial community under the 200% CL treatment was enhanced by 2.58 to 3.44%, but under the 100 CL % and 300 CL treatments was decreased by 2.38 to 8.76%. And the Simpson of bacteria community under nitrogen fertilizer addition was increased by 1.08 to 5.85%.

Under the same nitrogen fertilizer addition levels, the Chao1 of the bacteria community under 100% CL and 200% CL of AB treatments were enhanced by 19.84 and 22.31% compared to U treatments, but Chao1 of the bacteria community under 300% CL of AB treatment was reduced by 51.87% compared to U treatment. And the Shannon and Simpson of the bacteria community at 100% CL and 200% CL of AB treatments were reduced by 0.83 to 1.55% compared to U treatments, but the Shannon and Simpson of the bacteria community at 300% CL of AB treatment was enhanced by 1.52 to 5.06% compared to U treatments.

Under the U treatment condition, a trend of the decreasing and then increasing of the Chao1 of the bacteria community was observed, which was opposite to the changing trend of Shannon and Simpson with increasing of nitrogen fertilizer addition levels. With increasing of AB addition levels, the Chao1 of the bacteria community displayed a decreasing trend, and the Shannon of the bacteria community showed an increasing and then decreasing trend, and an increasing trend of the Simpson of the bacteria community was observed.

Nonmetric multidimensional scaling (NMDS) indicated that the bacterial community beta diversity was significantly altered by AB fertilizer application but was not significantly changed by U treatment compared to CK treatment (Figure 3A). The bacterial community beta diversity was significantly altered by 100% CL (U1 and AB1) and 300% CL (U3 and AB3) treatment but was not significantly changed by 200% CL (U2 and AB2) compared to CK treatment (Figure 3B). Under the nitrogen fertilizer types, nitrogen fertilizer levels, and combination with the type and level of the nitrogen fertilizer application, the explanation of bacterial beta diversity was calculated as 20, 34, and 59%, respectively, *via* the method of permutational multivariate analysis of variance.

### 3.4. The effect of bacterial on the weathering of purple mudstone particles under nitrogen fertilizer application

After 120 days of the laboratory experiment, the mineral elements’ content of purple mudstone particles was measured to calculate the chemical index of alteration (*CIA*), the chemical index of weathering (*CIW*), the chemical proxy of alteration (*CPA*), and the modified CIA (*CIX*) (Table 2). The *CIA*, *CIW*, *CPA*, and *CIX* of the purple mudstone particles after 120 days of incubation with fertilizer application were enhanced by 1.22% ~ 9.85, 47.01% ~ 70.23, 1.07% ~ 6.47, and 0.15% ~ 6.08%, respectively, compared to before the incubation treatments. Compared with the CK treatment, the *CIA*, *CIW*, and *CPA* of purple mudstone particles under nitrogen fertilizer application treatments were enhanced by 0.61 to 8.52%, 1.75 to 15.80%, and 1.19 to 5.35%, respectively, and the *CIX* of purple mudstone particles under U2 and U3 treatment were reduced by 0.18 and 0.27%, respectively, but other treatments were enhanced by 1.86 to 5.63%. Under the same nitrogen fertilizer level application, the weathering indices (*CIA*, *CIW*, *CPA*, and *CIX*) of the purple mudstone particles under AB treatment were higher than that under U treatments. Additionally, weathering indices (*CIA*, *CIW*, *CPA*, and *CIX*) of the purple mudstone particles under both the U and AB treatments showed a decreasing trend with increasing nitrogen fertilizer application levels.

**Table 2:** The chemical index of alteration response to nitrogen fertilization addition after 120 days in the laboratory.

Treatment	*CIA*	*CIW*	*CPA*	*CIX*
Original rock	61.8 ± 0.2	34.9 ± 0.4	83.0 ± 0.3	70.8 ± 0.2
CK	62.6 ± 0.8a	52.8 ± 0.9ab	83.9 ± 0.4a	71.1 ± 0.8ab
U1	65.6 ± 0.5bcd	56.3 ± 1.5b	86.2 ± 0.5bcd	73.3 ± 0.3abc
U2	63.1 ± 1.2ab	52.7 ± 1.3ab	85.1 ± 0.7abc	71.0 ± 1.1a
U3	62.9 ± 0.7ab	51.6 ± 1.0a	84.8 ± 0.4ab	70.9 ± 0.5a
AB1	67.9 ± 0.7d	56.9 ± 0.8b	87.6 ± 0.4d	75.1 ± 0.6c
AB2	66.0 ± 0.8 cd	53.3 ± 2.2ab	86.7 ± 0.4 cd	73.4 ± 0.7bc
AB3	64.6 ± 1.0abc	52.9 ± 1.3ab	85.6 ± 0.8abc	72.4 ± 0.8ab

ANOVA was conducted followed by the Duncan test for multiple comparisons. Different lowercase letters indicate that there were significant differences among all treatments (*p* < 0.05).The weathering indices of the purple mudstone before incubation (original rock) and after incubation (CK, U1, U2, U3, AB1, AB2, and AB3) were displayed in Table 2, and the indicators to characterize the weathering of purple mudstone included the Chemical index of alteration (CIA-Nesbitt and Young, 1982), Chemical index of weathering (CIW- Harnois, 1988), Chemical proxy of alteration (CPA- Buggle et al., 2011), and Modified CIA (CIX-Garzanti et al., 2014), which were calculated and showed in Table 2.

Stepwise regression analysis was used to analyze the effect of the dominant bacterial species at the genus level and environmental factors on the weathering indices (*CIA*, *CIW*, *CPA*, and *CIX*) of purple mudstone particles in this study (Table 3). The stepwise regression analysis indicated that Nitrobacter (*Nitrolancea*) and Phosphorus-dissolving bacteria (*Massilia*) play the main roles in the weathering of the purple mudstone particles, and the N-NO_3_^−^ was measured as a key environmental factor during the purple mudstone particles’ weathering process. The proportions of *Nitrolancea*, *Massilia*, and N-NO_3_^−^ that affected the weathering indices (*CIA*, *CIW*, *CPA*, and *CIX*) of J_3_p purple mudstone particles were calculated to be 36, 12, and 35% by the stepwise regression analysis, respectively (Table 3).

**Table 3:** The weathering indices and the content-dissolved organic carbon and nitrogen of the J_3_p purple mudstone fragaments’ response to the bacteria of the top 10 at the genus level after 120 days of nitrogen fertilizer addition in the laboratory.

Dependent variable	Enter variable	Remove variable	Parameter variable	Partial *R*^2^	Model *R*^2^	*p*
Weathering indices	Equation intercept		−0.68			
	*Nitrolancea*		5.89	0.36	0.36	0.00
		*Faecalibacterium*	0.04	0.00	0.36	0.83
	*Massilia*		5.33	0.12	0.48	0.03
		*Domibacillus*	−0.04	0.00	0.48	0.81
		*Sericytochromatia*	0.10	0.00	0.48	0.65
		*Lysobacter*	−0.05	0.00	0.48	0.78
		*Sphingomonas*	−0.05	0.00	0.48	0.80
		*Nitrosospira*	0.47	0.07	0.48	0.06
		*Luteimonas*	−0.21	0.00	0.48	0.59
		*Nocardioides*	−0.03	0.00	0.48	0.87
Weathering indices	Equation intercept		−0.44			
	N-NO_3_^−^		1.44	0.35	0.35	0.00
		pH	0.24	0.00	0.35	0.76
		DOC	−0.14	0.00	0.35	0.47
		TDN	−0.04	0.00	0.35	0.84
		Net nitrification rate	−7.43	0.00	0.35	0.98
		N-NH_4_^+^	0.00	0.00	0.35	0.41

The dependent variable (weathering indices) was obtained by reducing dimensionality (Principal component analysis) of CIA, CIW, CPA, and CIX. And based on the multivariable linear regression model, the effect of dominated species of bacteria at genus and the environment factors (pH, dissolved organic carbon (DOC), total dissolved nitrogen (TDN), N-NH4+, and N-NO3−) on weathering indices were calculated, and key factor were separated from all factors. In addition, this model’s results were applied to construct a structural equation model.

## 4. Discussion

### 4.1. The effect of nitrogen application on the characteristics of bacterial community diversity

Nitrogen fertilizer application altered the alpha and beta diversities of the bacterial community (Figures 2, 3), and the effect of the nitrogen fertilizer addition levels on the alpha and beta diversities of the bacterial community was more prominent. The bacterial community was controlled by the environmental factors and interaction relationships of bacteria (Zhang et al., 2017; Nie et al., 2018; Sun et al., 2020). In terms of environmental factors, the bacterial community diversity was determined by the pH of the soil environment (Rousk et al., 2010; Zhang et al., 2017). Previous studies have demonstrated that the number of bacterial species showed a trend of increasing and then decreasing with increasing pH of the soil environment in alkaline soil (pH range 7.43 ~ 8.66), and the optimal environmental conditions for bacterial growth were observed to be pH 8.04 (Shi et al., 2021). Another study also implied that the Shannon index increased following a decreasing trend with increasing soil pH (pH range 4.31–8.31) in a 7-year long-term study, and the soil pH was significantly altered by fertilizer addition (Zhang et al., 2017). This may be due to the poorer tolerance of the bacteria in the alkaline environment (Bárcenas-Moreno et al., 2016) or the narrower pH for optimal growth of bacteria (Rousk et al., 2010). In this study, the effect of the nitrogen fertilizer application levels on the pH of the soil environment was higher than that of the nitrogen fertilizer application types (Table 1), and the pH (range 7.88–9.46) displayed an increasing trend with increasing nitrogen fertilizer addition levels, which resulted in the dominant species of bacteria at the genus level significantly altering with increasing pH (Supplementary Figure S2). The relative abundance of the *Nitrolancea* and *Luteimonas* displayed a decreasing trend but other dominant species in the genus observed an increasing trend with the pH increasing (Supplementary Figure S2). Based on the polynomial fitting model analysis, it was found that with an increase in pH, the Chao1 of the bacterial community exhibits a nonlinear trend (*R*^2^ = 0.09, *p* = 0.39) of decreasing and then increasing (Supplementary Figure S3a), while the Simpson (*R*^2^ = 0.24, *p* < 0.05) and Shannon (*R*^2^ = 0.22, *p* < 0.05) of the bacterial community showed nonlinear characteristics of first increasing and then decreasing (Supplementary Figures S3b,S3c), which was caused by changes in dominant bacterial species. In addition, the alpha diversities displayed nonlinear characteristics of increasing and then decreasing with increasing nitrogen fertilizer addition levels in previous studies (Zhang et al., 2008; Li et al., 2019; He et al., 2021). However, another environmental factor in the bacterial community was determined by the N-NH_4_^+^ content in the soil (Nie et al., 2018). The N-NH_4_^+^ content increased with increasing nitrogen fertilizer addition levels, but the N-NH_4_^+^ content was not different under the different nitrogen fertilizer addition types at the same fertilizer levels (Table 1). The high N-NH_4_^+^ concentration in the soil was toxic to some bacterial groups, and the low N-NH_4_^+^ concentration in the soil promoted bacterial growth (Zhou et al., 2017) in a previous study. According to the polynomial fitting model analysis, the alpha diversities **(Chao1, Shannon, and Simpson)** of the bacterial community showed nonlinear characteristics with increasing N-NH_4_^+^, and the Chao1 (*R*^2^ = 0.19, *p* = 0.06) of the bacterial community displayed a decreasing and following increasing trend (Supplementary Figure S3d), while the Simpson (*R*^2^ = 0.23, *p* < 0.05) and Shannon (*R*^2^ = 0.14, *p* = 0.09) of the bacterial community (Supplementary Figures S3e,S3f) showed increasing first and then decreasing. Additionally, a significantly negative relationship was calculated between pH (*R*^2^ = 0.80, *p* < 0.05), N-NH_4_^+^ (*R*^2^ = 0.21, *p* < 0.05), and beta diversity of the bacterial community (Supplementary Figures S3g,S3h). Based on the above analysis in terms of environmental factors, changes in the pH and N-NH_4_^+^ in the soil environment were induced by the application of different nitrogen fertilizer levels, which was one of the main reasons inducing the succession of the dominant species in the soil environment, thereby inducing differences in the structural alpha and beta diversities of the bacterial community.

In terms of the interrelationship of the bacterial communities, antagonistic and synergistic effects were widely found between bacterial communities (Meidute et al., 2008; Cai et al., 2019). Another previous study showed that the antagonistic effect of inorganic fertilizer application treatment was observed to have a main role in the bacterial community compared to no fertilizer addition and organic fertilizer treatment (Sun et al., 2020). Previous studies reported that the toxic effector proteins secreted by the type VI secretion system (T6SS) were one of the main ways that the antagonistic effect was induced between bacterial communities (Trunk et al., 2018), and the toxic effector proteins were transported into the nucleus of neighboring fungi or bacteria to promote bacterial or fungal lethality (Tang et al., 2018; Jurėnas and Journet, 2021). The dominant species may be altered by antagonistic effects of bacteria under different nitrogen fertilizer applications, thereby affecting the beta diversity of bacteria. In this study, compared to the dominant species structure in the CK treatment, smaller differences in the dominant species structure under different nitrogen fertilizer addition types were observed, and larger differences in the dominant species structure under different nitrogen fertilizer addition levels were observed, which resulted in a higher effect of nitrogen fertilizer addition levels on bacterial beta diversity than nitrogen fertilizer addition types. The difference in nitrogen fertilizer addition treatment may be due to the composition of the bacterial community being codetermined by the adaptation of the bacteria to the soil environment change (Bárcenas-Moreno et al., 2016) and the interrelationship between species (Meidute et al., 2008), thereby affecting the beta diversity of the bacterial community. In addition, a global meta-analysis implied that the beta diversity of the bacterial community increased with increasing nitrogen fertilizer addition levels (Zhou et al., 2022). The antagonistic effects of the bacteria at nitrogen fertilizer application may be one of the factors that induced the dominant species structure alteration in this study, thereby promoting the change in the beta diversity of bacteria.

### 4.2. The effect of nitrogen application on the weathering of purple mudstone particles

**Figure 4:**
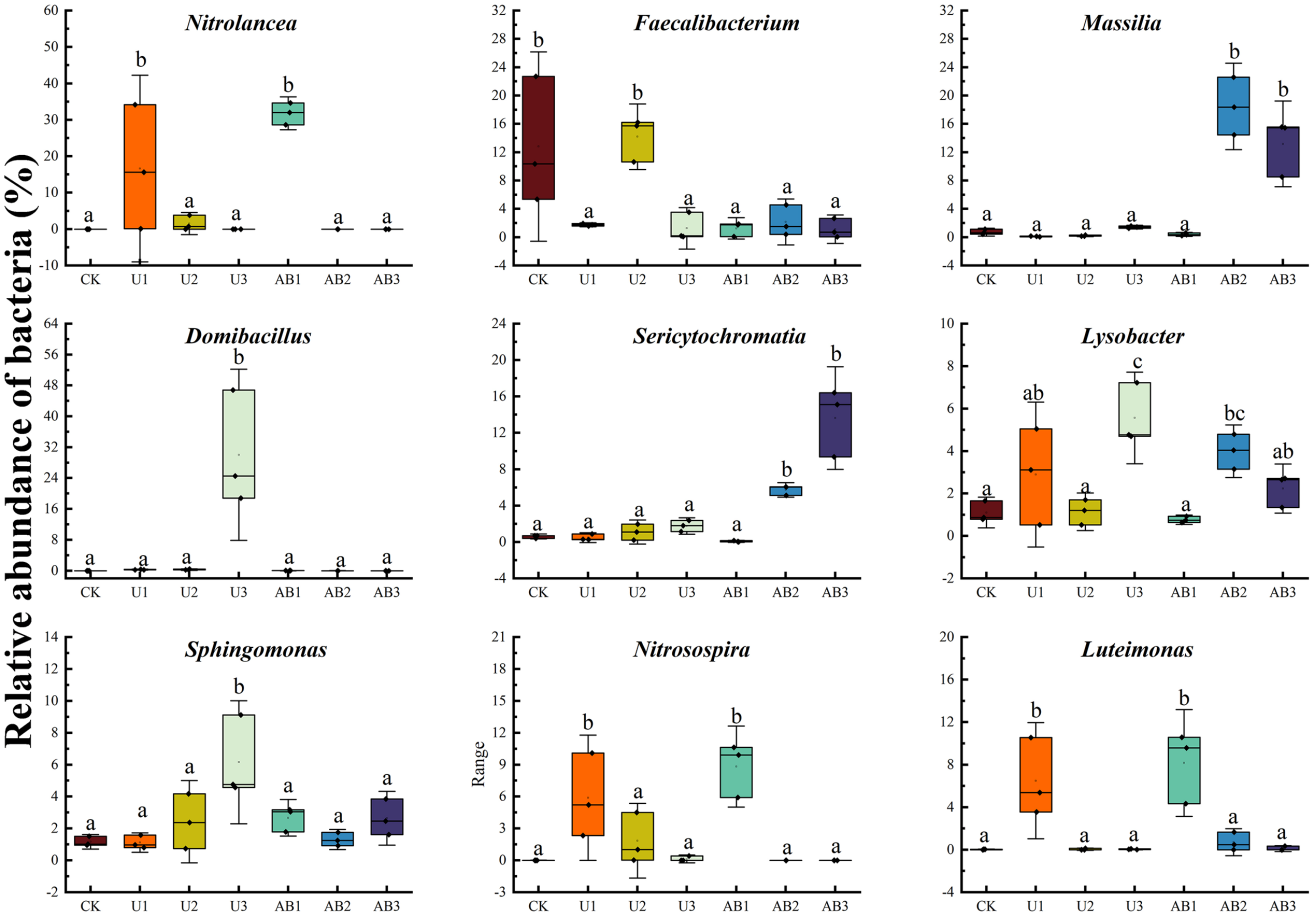
Bacterial species at subordinate levels of different nitrogen fertilization treatments after 120 days in the laboratory. The no fertilizers (CK), urea of 280 N kg∙ha^−1^ (U1), urea of 560 N kg∙ha^−1^ (U2), urea of 840 N kg∙ha^−1^ (U3), ammonium bicarbonate of 280 N kg∙ha^−1^ (AB1), ammonium bicarbonate of 560 N kg∙ha^−1^ (AB2), and ammonium bicarbonate of 840 N kg∙ha^−1^ (AB3) were observed. Different lowercase letters indicate that there were significant differences among all treatments (*p* < 0.05).

This result indicated that the weathering indices (*CIA*, *CIW*, *CPA*, and *CIX*) of the purple mudstone particles were promoted by nitrogen fertilizer addition compared with the CK treatment and were measured with a decreasing trend with increasing nitrogen fertilizer levels applied under the same nitrogen fertilizer types (Table 2). Our previous studies have confirmed that acid input will accelerate purple rock weathering (Zhao et al., 2021). More specifically, the H^+^ was expanded by alkaline anions in the soil solution (Rice and Herman, 2012) and the exchangeable base ions in clay minerals (Warby et al., 2009). After the above reaction, the clay minerals were decomposed by residual H^+^ to accelerate the base ion release from the clay minerals (Tabatabai and Olson, 1985), thereby promoting the weathering of rock. Additionally, the water-soluble Ca^2+^ was measured and displayed a decreasing trend with the nitrogen fertilizer addition levels increasing in this study (Supplementary Table S3), and a significant positive correlation was calculated between the content of water-soluble Ca^2+^ and the weathering indices (Supplementary Figure S4a) but a negative correlation of the content of CaO in clay minerals and weathering indices was observed (Supplementary Figure S4b). The previous studies implied that the weathering carbonate was accelerated by nitrogen fertilizer addition, and the leaching amount of the base ion was enhanced due to the lack of a buffering reaction in carbonate-free soil, however, the soil acidification after nitrogen fertilizer application was offset by the buffering reaction that occurred in carbonated soils (Gandois et al., 2011). And another study indicated that an increasing trend of weathering rate of carbonate, CO_2_ concentration, Ca^2+^, Mg^2+^, and NO_3_^−^ was measured after nitrogen fertilizer addition, which was attributed to the release of protons from the nitrification reaction (Song et al., 2017). This provides ample evidence that nitrogen fertilizer application affects the weathering indices of purple mudstone particles that are rich in Calcite (Supplementary Table S1). Moreover, the H^+^ by nitrification of N-NH_4_^+^ was one of the main sources of H^+^ in the soil environment, yet the relative abundance of *Nitrolancea*, a nitrite-oxidizing bacteria (NOB) (Sorokin et al., 2014; Jeong and Bae, 2021), showed a decreasing trend with increasing nitrogen fertilizer addition levels (Figures 1, 4), which resulted in the nitrification of N-NH_4_^+^ decreasing with increasing nitrogen fertilizer addition levels. The main reason for this phenomenon may be due to the high N-NH_4_^+^ concentration in the soil being toxic to some bacterial groups, and the low N-NH_4_^+^ concentration in the soil promoted bacterial growth (Zhou et al., 2017). The growth of bacteria with nitrification was inhibited by the concentration of N-NH_4_^+^ with increasing nitrogen fertilizer application levels. This results in a reduction in the H^+^ produced by nitrification of N-NH_4_^+^ and therefore a reduction trend in the weathering of the purple mudstone particles with increasing nitrogen fertilizer application levels.

**Figure 5:**
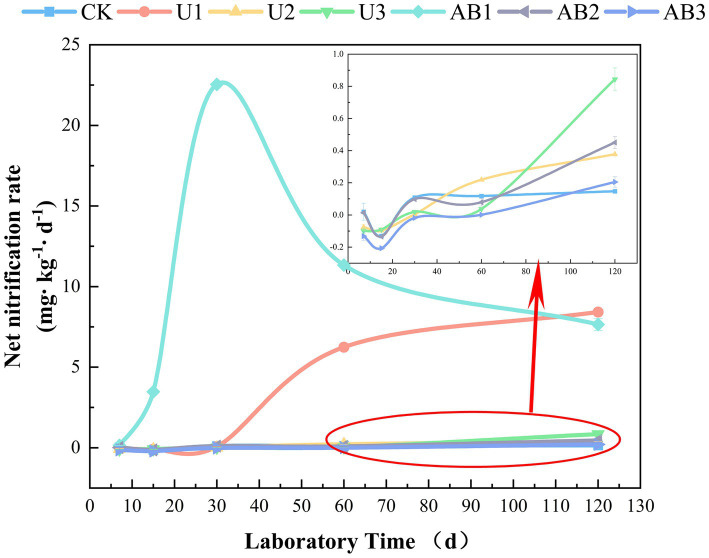
The net nitrification rate during the laboratory under nitrogen fertilizer addition. The no fertilizers (CK), urea of 280 N kg∙ha^−1^ (U1), urea of 560 N kg∙ha^−1^ (U2), urea of 840 N kg∙ha^−1^ (U3), ammonium bicarbonate of 280 N kg∙ha^−1^ (AB1), ammonium bicarbonate of 560 N kg∙ha^−1^ (AB2), and ammonium bicarbonate of 840 N kg∙ha^−1^ (AB3) were observed.

The weathering indices (*CIA*, *CIW*, *CPA*, and *CIX*) of the purple mudstone particles with AB application was higher than that with U application under the same nitrogen fertilizer levels but did not reach a significant level (*p* > 0.05), and the weathering indices (*CIA*, *CIW*, *CPA*, and *CIX*) of the purple mudstone particles were reduced with the nitrogen fertilizer addition level increasing (Table 2). The weathering indices (*CIA*, *CIW*, *CPA*, and *CIX*) of the purple mudstone particles were controlled by H^+^, from net nitrification of *Nitrolancea*. And net nitrification rate was significantly reduced (Supplementary Figure S5) with the nitrogen fertilizer addition level increasing. The average nitrification rate at 200% CL and 300% CL can be ignored because the average nitrification rate was observed by −0.03 mg∙kg^−1^∙d^−1^ to 0.14 mg∙kg^−1^∙d^−1^ (Figure 5), therefore, the nitrification of *Nitrolancea* at 100% CL was one of the main factors to affect the weathering of the purple mudstone particles. Additionally, *Massilia* may be another key bacterial in the weathering of purple mudstone particles. In this study, under the 200% CL and 300% CL nitrogen fertilizer treatments, *Massilia* was observed in the AB treatment but not in the U treatment. According to previous research, *Massilia* has a dissolved phosphorus function (Zheng et al., 2017). The phosphorus dissolution process of bacteria may promote the release of calcium and magnesium, which may affect rock weathering. In addition, a previous study indicated that highly effective Al solubilizers from *Bacillus* secretion promote the release of Al from surface rocks (Wang et al., 2017). Another study also reported that acetic acid was found in the metabolites of mucilaginous *Bacillus*, and the complexation of acetic acid was one of the main mechanisms that accelerated silicate clay mineral weathering (Mo and Lian, 2011). These studies provide some strong evidence that the secretions of bacteria promote rock weathering. The effects of nitrification of N-NH_4_^+^ or phosphorus dissolution of *Massilia* were shown to be stronger with the AB treatment than with the U treatment, thereby indicating that the weathering indices (*CIA*, *CIW*, *CPA*, and *CIX*) of the purple mudstone particles in the AB treatment were greater than that in the U treatment.

**Figure 6:**
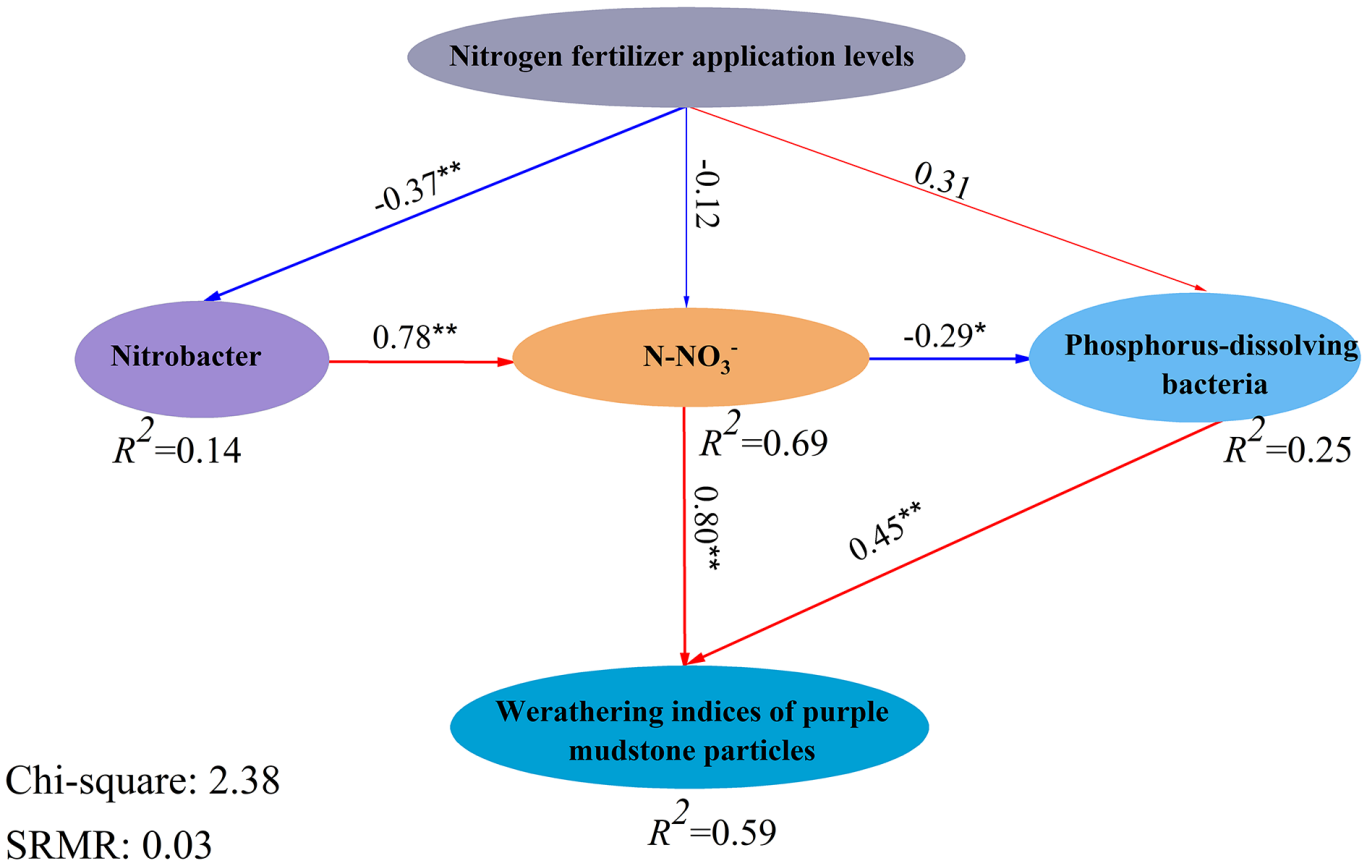
The model of the bacteria on weathering indices (*CIA*, *CIW*, *CPA*, and *CIX*) of J_3_p purple mudstone particles under different nitrogen fertilizer additions. The names inside the boxes are measured variables, and the labels below the circles are latent variables. The numbers associated with the arrows are path coefficients. The percentages inside the boxes refer to the variance explained by the Model (*R*^2^). The significance correlations (*p* < 0.05) and extremely significant correlations (*p* < 0.01) of the physicochemical properties are expressed in “^*^” and “^**^,” respectively.

The PLS-SEM indicated that the explanation of the effect of bacteria on the weathering of purple mudstone particles was calculated as 59% after nitrogen fertilizer application, and the nitrogen fertilizer addition levels had a negative effect on Nitrobacter (*p* < 0.01) and N-NO_3_^−^ (*p* = 0.50) but had a positive effect on Phosphorous-dissolving bacteria (*p* = 0.16). And Nitrobacter had an extremely significant positive effect on the N-NO_3_^−^ (*p* < 0.01), while the N-NO_3_^−^ had a negative effect on Phosphorous-dissolving bacteria (*p* < 0.05) (Figure 6). Noteworthy is the opposite positive effect of the N-NO_3_^−^ (*p* < 0.01), and Phosphorous-dissolving bacteria (*p* < 0.01) on weathering indices (*CIA*, *CIW*, *CPA*, and *CIX*) (Figure 6). Based on the above analysis, the H^+^ from nitrification of Nitrobacter (*Nitrolancea*) (Sorokin et al., 2014; Jeong and Bae, 2021) and the phosphorus dissolution of Phosphorous-dissolving bacteria (*Massilia*) (Zheng et al., 2017) may be the main processes affecting the weathering of purple mudstone particles, which was confirmed by the PLS-SEM calculation. The relative abundances of *Nitrolancea* and *Massilia* were significantly altered by different nitrogen fertilizer additions in this study (Figure 6). *Nitrolancea* was observed in the 100% CL treatment but was not detected in the 200% CL and 300% treatment, and *Massilia* was observed in the 200% CL and 300% treatment but was not detected in the 100% CL treatment. In summary, nitrogen fertilizer addition, especially AB application, can promote the weathering of purple mudstone particles in this study. Nitrification (*Nitrolancea*) was the main pathway for nitrogen fertilizer application affecting the weathering of purple mudstone particles at conventional levels (100% CL), but phosphorus dissolution of *Massilia* may be the main pathway for nitrogen fertilizer affecting the weathering of purple mudstone particles at excessive application (200% CL and 300% CL).

## 5. Conclusion

At the same fertilization levels, the weathering indices (*CIA*, *CIW*, *CPA*, and *CIX*) of purple mudstone particles under the AB treatment were higher than that under the U treatment, and a reducing trend was observed with increasing nitrogen fertilizer levels under the same nitrogen fertilizer application types. Compared with the CK treatment, the *CIX* of purple mudstone particles at U2 and U3 treatments were reduced by 0.18, and 0.27%, respectively, however, the weathering indices (*CIA*, *CIW*, *CPA*, and *CIX*) of purple mudstone particles at other fertilizer treatments were enhanced by 0.61 to 15.80%. Nitrogen fertilizer application significantly altered the diversity of the bacterial community. Specifically, the alpha diversities of the bacterial community appeared to increase first and then decline as the nitrogen fertilizer levels increased, and the effect of the nitrogen fertilizer application levels (*R^2^* = 0.34) on beta diversity was higher than that of the nitrogen fertilizer type (*R^2^* = 0.20). Compared with the CK treatment, the beta diversity of bacteria under the AB treatment was significantly changed, while it was not significantly altered under the U treatment. The beta diversity was significantly enhanced compared to the CK treatment in the 100% CL and 300% CL treatments, but it was not significantly different from the CK treatment in the 200% CL treatment. The impact proportions of *Nitrolancea*, *Massilia*, and N-NO_3_^−^ on the weathering indices (*CIA*, *CIW*, *CPA*, and *CIX*) of J_3_p purple mudstone particles were calculated to be 36, 12, and 35% by the stepwise regression analysis, respectively. Notably, the result of the calculations for the stepwise regression analysis and the structural equation model maintained a high degree of consistency. Additionally, the result of the structural equation model implied that the nitrification of Nitrobacter (*Nitrolancea*) and the phosphorus dissolution of Phosphorous-dissolving bacteria (*Massilia*) are the main drivers of the dominant weathering process of the J_3_p purple mudstone particles (*R^2^* = 0.59).

## Data availability statement

The raw data supporting the conclusions of this article will be made available by the authors, without undue reservation.

## Author contributions

CL, MF, XW, XL, GZ, GL, and JZ contributed to the study’s conception and design. Material preparation, data collection, and analysis were performed by CL, XW, and XL. The first draft of the manuscript was written by CL. GL, GZ, MF, and JZ commented on previous versions of the manuscript. All authors contributed to the article and approved the submitted version.

## Funding

This work was supported by the National Natural Science Foundation of China (No.42007002), the Yunnan Basic Research Project-general projects (No. 202101AT070220), and the Doctoral Research Foundation of Yunnan Agricultural University (No. KY2018-26).

## Conflict of interest

The authors declare that the research was conducted in the absence of any commercial or financial relationships that could be construed as a potential conflict of interest.

## Publisher’s note

All claims expressed in this article are solely those of the authors and do not necessarily represent those of their affiliated organizations, or those of the publisher, the editors and the reviewers. Any product that may be evaluated in this article, or claim that may be made by its manufacturer, is not guaranteed or endorsed by the publisher.

## Footnotes

^1^ https://docs.qiime2.org/2019.4/tutorials/

^2^ https://www.ncbi.nlm.nih.gov/sra/PRJNA881222
