# Supplementary material for: Effects of nitrogen fertilizers on the bacterial community diversity and the weathering of purple mudstone in Southwest China
Source: Front Microbiol. 2023 Jun 29;14:1164826. doi: 10.3389/fmicb.2023.1164826 (PMC10341161; doi:10.3389/fmicb.2023.1164826)
Supplement: Supplementary file 1 [file Data_Sheet_1.pdf]

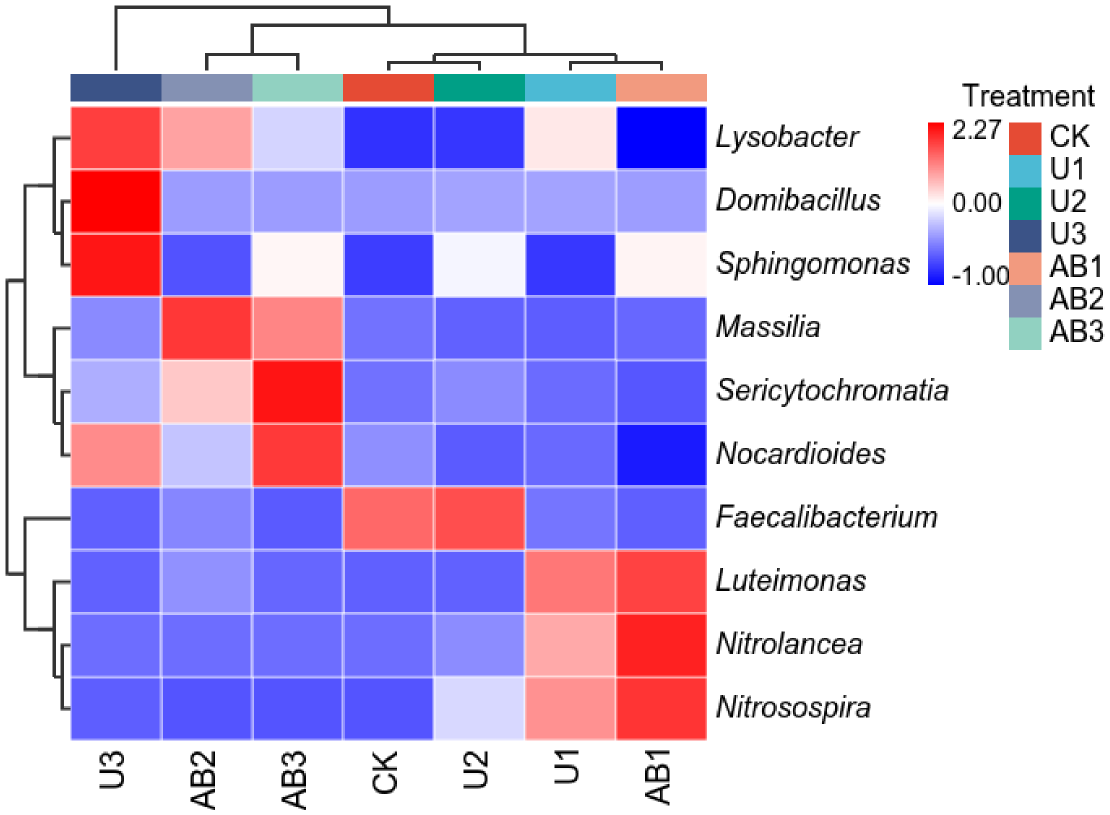

Figure S1. Bacterial signature species at subordinate levels of different nitrogen fertilization treatments after 120 days in the laboratory. The no fertilizers (CK), urea of 280 N kg·ha^{-1}(U1), urea of 560 N kg·ha^{-1} (U2), urea of 840 N kg·ha^{-1}(U3), ammonium bicarbonate of 280 N kg·ha^{-1}(AB1), ammonium bicarbonate of 560 N kg·ha^{-1} (AB2), and ammonium bicarbonate of 840 N kg·ha^{-1} (AB3) were observed.

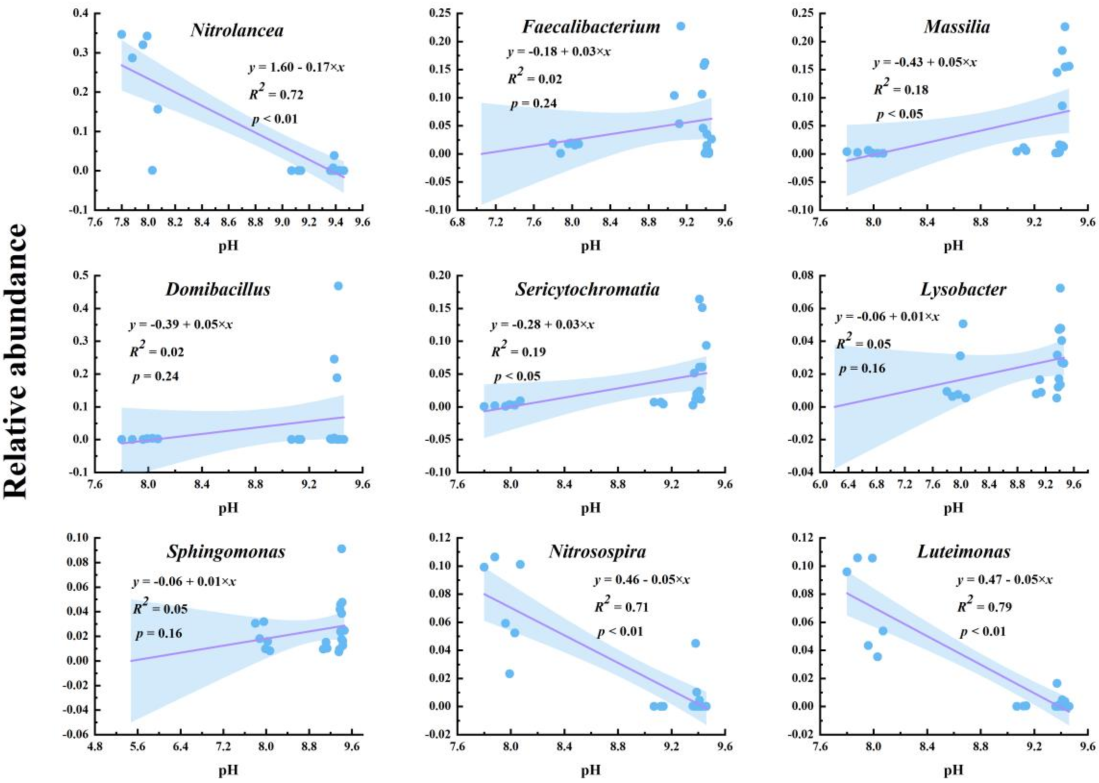

Figure S2. The effect of the pH on the main bacterial abundance under nitrogen fertilizer application

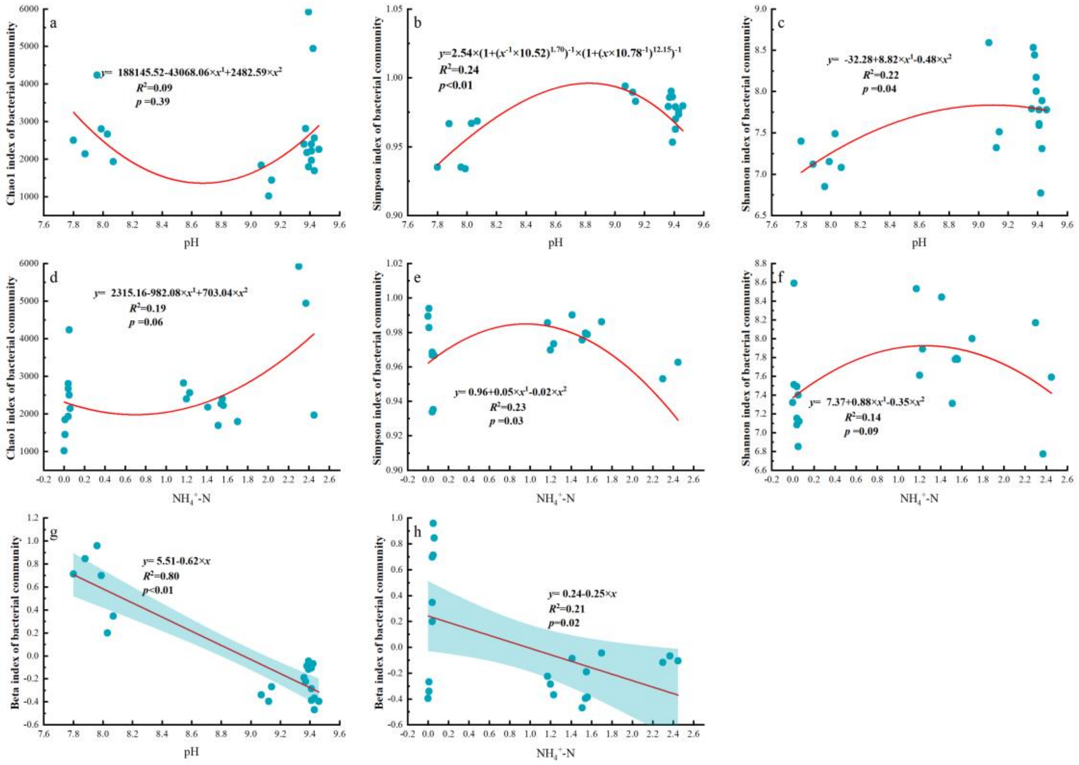

Figure S3. The non-linear relationship was calculated between the pH (a, b, and c), N-NH$_4^+$(d, e, and f), and alpha diversities (Chao1, Simpson, and Shannon) of the bacteria by the polynomial fitting model and the linear relationship was calculated between the pH (g), N-NH$_4^+$ (h), and beta diversity (NMDS1 Score) of the bacteria by linear fitting analysis

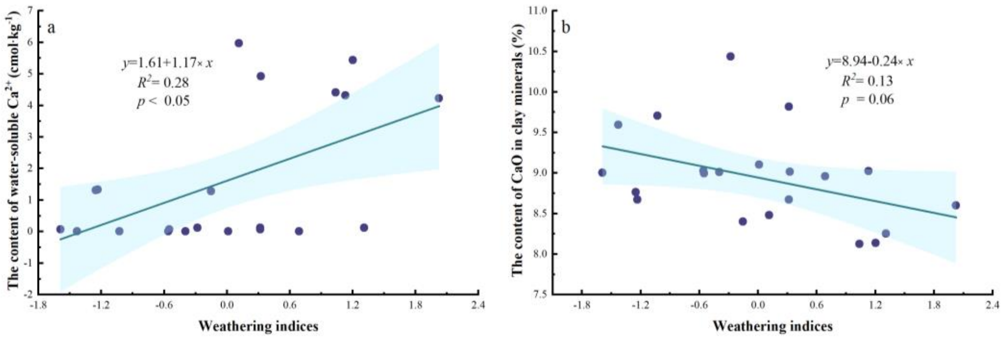

Figure S4. The linear relationship was calculated between the content of water-soluble Ca^{2+} (a) and the content of CaO in clay minerals (b), and the weathering indices, respectively. And weathering index is the score calculated by the Chemical index of alteration (*CIA*), the Chemical index of weathering (*CIW*), the Chemical proxy of alteration (*CPA*), and the Modified CIA (*CIX*) based on principal component analysis.

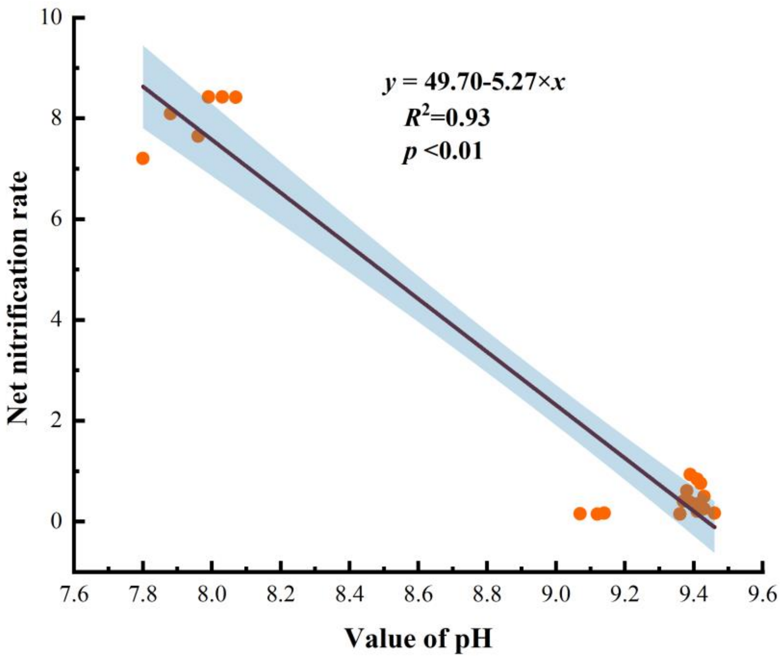

Figure S5. The linear relationship calculated between the pH and net nitrification rate by linear fit analysis
