# Supplementary material for: Effects of nitrogen fertilizers on the bacterial community diversity and the weathering of purple mudstone in Southwest China
Source: Front Microbiol. 2023 Jun 29;14:1164826. doi: 10.3389/fmicb.2023.1164826 (PMC10341161; doi:10.3389/fmicb.2023.1164826)
Supplement: Supplementary file 3 [file Table_1.docx]

**Supplemental Tables**

**Table S1: The main components of clay minerals of the J_3_p purple mudstone particles**

| Montmorillonite | Illite | Kaolinite | Chlorite | Vermiculite | Mica |
| --- | --- | --- | --- | --- | --- |
| - | 9 | 3 | 8 | 9 | 8 |
| Potassium feldspar | Plagioclase | Calcite | Dolomite | Quartz |  |
| 1 | 11 | 12 | 3 | 34 |  |

**Table S2: The main oxide changes of the J_3_p purple mudstone particles before and after the incubation experiment (%)**

| Oxide | CK | U1 | U2 | U3 |
| --- | --- | --- | --- | --- |
| SiO_2_ | 53.44±0.60 | 49.47±1.95 | 52.40±1.01 | 53.24±0.10 |
| Al_2_O_3_ | 22.37±0.71 | 25.33±1.23 | 22.46±0.92 | 22.02±0.46 |
| Fe_2_O_3_ | 7.04±0.28 | 8.04±0.41 | 7.88±0.29 | 7.47±0.13 |
| K_2_O | 4.42±0.12 | 4.77±0.25 | 4.84±0.07 | 4.71±0.01 |
| Na_2_O | 2.61±0.09 | 2.46±0.05 | 2.38±0.06 | 2.39±0.04 |
| CaO | 8.61±0.11 | 8.54±0.26 | 8.89±0.11 | 9.21±0.19 |
| MgO | 1.51±0.23 | 1.38±0.21 | 1.15±0.20 | 0.97±0.20 |
| Oxide | AB1 | AB2 | AB3 | Original rock |
| SiO_2_ | 50.59±1.07 | 50.55±0.65 | 52.15±0.52 | 54.79±2.64 |
| Al_2_O_3_ | 25.34±0.87 | 23.86±0.77 | 23.33±0.64 | 17.88±1.41 |
| Fe_2_O_3_ | 7.68±0.92 | 7.84±0.13 | 7.11±0.39 | 4.28±0.11 |
| K_2_O | 4.43±0.01 | 4.59±0.03 | 4.58±0.05 | 3.42±0.01 |
| Na_2_O | 2.18±0.03 | 2.22±0.03 | 2.38±0.09 | 2.23±0.00 |
| CaO | 8.58±0.26 | 9.50±0.65 | 9.26±0.23 | 16.32±0.12 |
| MgO | 1.20±0.16 | 1.44±0.19 | 1.19±0.21 | 1.08±0.09 |

Notes: The main oxide of the J_3_p purple mudstone before incubation (original rock) and after incubation (no fertilizers (CK), urea of 280 N kg∙ha^-1^(U1), urea of 560 N kg∙ha^-1^ (U2), urea of 840 N kg∙ha^-1^(U3), ammonium bicarbonate of 280 N kg∙ha^-1^(AB1), ammonium bicarbonate of 560 N kg∙ha^-1^ (AB2), and ammonium bicarbonate of 840 N kg∙ha^-1^ (AB3) were displayed in Table S2. The same as below.

**Table S3: The Ca^2+^ of water-soluble of the J_3_p purple mudstone particles after the incubation experiment (cmol**∙**kg^-1^)**

| Treatment | Content of water-soluble Ca^2+^ |
| --- | --- |
| CK | 1.31±0.01b |
| U1 | 5.44±0.30d |
| U2 | 0.07±0.00a |
| U3 | 0.01±0.00a |
| AB1 | 4.32±0.05c |
| AB2 | 0.12±0.00a |
| AB3 | 0.01±0.00a |
